# ER stress drives Lipocalin 2 upregulation in prostate cancer cells in an NF-κB-dependent manner

**DOI:** 10.1186/1471-2407-11-229

**Published:** 2011-06-07

**Authors:** Navin R Mahadevan, Jeffrey Rodvold, Gonzalo Almanza, Antonio Fernández Pérez, Matthew C Wheeler, Maurizio Zanetti

**Affiliations:** 1 The Laboratory of Immunology, Department of Medicine and Moores Cancer Center, University of California, San Diego, 9500 Gilman Drive, La Jolla, CA 92093-0815, USA

## Abstract

**Background:**

Tumor cells adapt to endoplasmic reticulum (ER) stress through a set of conserved intracellular pathways, as part of a process termed the unfolded protein response (UPR). The expression of UPR genes/proteins correlates with increasing progression and poor clinical outcome of several tumor types, including prostate cancer. UPR signaling can activate NF-κB, a master regulator of transcription of pro-inflammatory, tumorigenic cytokines. Previous studies have shown that Lipocalin 2 (Lcn2) is upregulated in several epithelial cancers, including prostate cancer, and recently Lcn2 was implicated as a key mediator of breast cancer progression. Here, we hypothesize that the tumor cell UPR regulates Lcn2 production.

**Methods:**

We interrogated Lcn2 regulation in murine and human prostate cancer cells undergoing pharmacological and physiological ER stress, and tested UPR and NF-κB dependence by using pharmacological inhibitors of these signaling pathways.

**Results:**

Induction of ER stress using thapsigargin (Tg), a canonical pharmacologic ER stress inducer, or via glucose deprivation, a physiologic ER stressor present in the tumor microenvironment, upregulates LCN2 production in murine and human prostate cancer cells. Inhibition of the UPR using 4-phenylbutyric acid (PBA) dramatically decreases Lcn2 transcription and translation. Inhibition of NF-κB in prostate cancer cells undergoing Tg-mediated ER stress by BAY 11-7082 abrogates Lcn2 upregulation.

**Conclusions:**

We conclude that the UPR activates Lcn2 production in prostate cancer cells in an NF-κB-dependent manner. Our results imply that the observed upregulation of Lipocalin 2 in various types of cancer cells may be the direct consequence of concomitant UPR activation, and that the ER stress/Lipocalin 2 axis is a potential new target for intervention in cancer progression.

## Background

Lipocalin 2 (Lcn2), otherwise known as neutrophil gelatinase-associated lipocalin (NGAL), is upregulated in several solid cancers, and has been shown to facilitate tumor progression. Yang *et al *[1] linked the elevated LCN2 levels found in breast cancer with increased tumor progression and metastasis and revealed its direct role in facilitating the epithelial-to-mesenchymal transition (EMT) in breast cancer cells. Importantly, a *Lcn2*-deficient mouse model of spontaneous breast cancer showed a decreased rate of cancer progression [2,3]. However, the regulation and function of Lcn2 in epithelial cancers remain unknown.

Lcn2 is a ligand for matrix metalloproteinase 9 (MMP9) in human neutrophils [4] and has innate immune function through the prevention of iron scavenging by bacterial siderophores [5]. Lipocalin 2 also binds a mammalian siderophore and traffics iron in mammalian cells [6-8]. The family of lipocalins, and particularly Lcn2, has been considered a marker of inflammatory processes such as obesity, atherosclerosis, and asthma [9]. Furthermore, NF-κB, a master regulator of inflammation, drives *Lcn2 *transcription in malignant and untransformed cells [2,10,11].

The tumor microenvironment differs markedly from that of normal tissues. Most notably, tumors lack a well-developed blood supply, which leads to decreased nutrient supply, low pH, and hypoxia. Compounding these extrinsic *noxae *are tumor-intrinsic stressors, which include oxidative stress, aberrant protein folding and glycosylation, viral infection, and defects in calcium homeostasis [12]. When un/misfolded proteins accumulate within the endoplasmic reticulum (ER) lumen due to tumor-intrinsic and extrinsic stressors, tumor cells experience ER stress. Like all eukaryotic cells, tumor cells adapt to ER stress by signaling through a conserved set of intracellular pathways known collectively as the unfolded protein response (UPR) [13]. The UPR is initiated by the ER chaperone molecule, Grp78, which, under conditions of ER stress, disassociates from three ER membrane-bound sensors (IRE1α, ATF6, and PERK), causing their activation. Downstream signaling cascades ameliorate ER stress via several mechanisms, including selective translation inhibition and upregulation of genes that encode enzymes that aid in protein folding, maturation, and degradation [14]. Involved in this homeostatic/regulatory cascade are two target genes, *Gadd34 *and *Chop*, that are associated with translational recovery and apoptosis, respectively [13].

Prostate cancer is the most prevalent cancer in men. Prostate cancer cells often gain resistance to early therapeutic interventions, and relapse is common (> 40%) [15]. Recently, the ability to mount the UPR has been linked to prostate tumorigenesis and cancer progression. For example, conditional deletion of *Grp78 *in the prostates of *Pten*-deficient mice abrogates prostate tumorigenesis [16]. Additionally, human prostate cancer cells express significantly more GRP78 than their benign counterparts, and increasing GRP78 expression correlates with recurrence and poor survival [17].

With the exception of work showing that the LCN2/MMP9 complex is more likely to be found in the urine of prostate cancer patients than controls [18], little is known about LCN2 in prostate cancer. Additionally, while ER stress and Lcn2 are both associated with cancer tumorigenesis, progression, and poor clinical outcome, to date there has been no mechanistic explanation for these correlations. We used an *in vitro *model of ER stress to test the hypothesis that ER stress and Lcn2 upregulation are causally linked in prostate cancer cells, as a first step in an effort to elucidate the broad role of constitutive ER stress in cancer cells. We demonstrate that the ER stress response in murine and human prostate cancer cells drives the production of *Lcn2 *in an NF-κB-dependent manner, and that diminishing the UPR dramatically decreases *Lcn2 *transcription and translation.

## Methods

### Cell Culture

TRAMP-C1 (TC1) cells were originally obtained from Dr. Andrew Weinberg (Oregon Health Science University). LNCaP and PC3 cells were purchased from American Type Culture Collection (ATCC). All lines were grown in complete RPMI-1640 medium supplemented with 10% heat-inactivated fetal calf serum (HyClone) and tested negative for mycoplasma (Lonza). All three cell lines were induced to undergo ER stress by the addition of 300 nM thapsigargin (Tg) (Alexis Biochemicals/Enzo Life Sciences, Plymouth, PA), 5 μg/mL of tunicamycin (Tun) (Sigma), or culture in medium lacking glucose, for indicated time points. Control cells were similarly treated with an equal volume of vehicle (100% ethanol or DMSO).

### Generation of TC1.pNGL and ER Stress Response/NF-κB Inhibition

The pNGL plasmid was graciously provided by Dr. T. Blackwell (Vanderbilt University, Nashville, TN). The pNGL plasmid contains a GFP/luciferase fusion gene driven by a minimal herpesvirus thymidine kinase promoter and eight decameric NF-κB binding sites (GGGGACTTTCC). TOP10 competent cells (Invitrogen) were transformed and selected on ampicillin-containing agar. Purified pNGL plasmid was obtained using the Wizard MiniPrep kit (Promega), and concentration and purity determined on a NanoDrop spectrophotometer (Thermo Scientific). TC1 cells were transfected in a 24 well plate with 2 μg DNA using the JetPEI reagent (Polyplus). Cells were initially selected in medium containing 800 μg/mL G418, and successful transfectants were maintained in 500 μg/mL G418.

For initial NF-κB kinetics experiments, cells were treated with Tg (300 nM) or vehicle (100% ethanol), or with 100 ng/mL LPS (Sigma) for the indicated time points. In ER stress response inhibition experiments, TC1.pNGL cells were treated for 8 h with Tg (300 nM) in the presence of 10 mM 4-phenylbutyric acid (PBA) (Sigma). In NF-κB inhibition experiments, TC1.pNGL cells (2.5 × 10^5^) were co-treated for 8 h with Tg (300 nM) and 25 μM Bay 11-7082 (EMD Chemicals). Live cells (7-AAD^-^) were gated and analyzed for NF-κB-driven EGFP reporter on a BDFacscalibur (BD Biosciences). Data was acquired and analyzed using BD CellQuest Pro and FlowJo (Tree Star) software.

### Quantitative RT-PCR

RNA was isolated from cells using the Nucleospin RNA II Kit (Macherey-Nagel). Concentration and purity of RNA was determined by analysis on a NanoDrop spectrophotometer (Thermo Scientific). cDNA was generated using the High Capacity cDNA Synthesis kit (Applied Biosystems) and quantitative PCR was performed on an ABI StepOne system using TaqMan reagents (Applied Biosystems) for 50 cycles. Target gene expression was normalized to *β*-*actin*, and analyzed using the -ΔΔCt relative quantification method. Results are expressed as fold change relative to the vehicle-treated control cells, whose gene expression profile did not differ from untreated cells. Validated FAM-labeled mouse *Il-23a *(Mm00518984_m1)*, Il-6 *(Mm99999064_m1), *Ddit3 *(Mm01135937_g1)*, Myd116 *(Mm00435119_m1)*, Hspa5 *(Mm00517691_m1), *Lcn2 *(Mm01324472_g1) and VIC-labeled mouse *β*-*actin *TaqMan primer/probe sets (Applied Biosystems) were used to analyze TC1 cDNA. Validated FAM-labeled human *IL-23A *(Hs00372324_m1)*, IL-6 *(Hs0098639_m1)*, HSPA5 *(Hs99999174_m1)*, LCN2 *(Hs00194353_m1), and VIC-labeled human *β*-*ACTIN *TaqMan primer/probe sets (Applied Biosystems) were used to analyze PC3 and LNCaP cDNA. Custom primers for human *CHOP *and *GADD34 *were synthesized (Applied Biosystems) using gene sequences with NCBI accession numbers NM_004083 and NM_014330, respectively. qPCR data was analyzed and statistical analysis performed using ABI StepOne software and GraphPad Prism software.

### Western Blot Analysis

After treatment, TC1 cells were washed in ice-cold PBS and re-suspended in cold lysis buffer containing 1% Triton X-100, 150 mM NaCl and 50 mM Tris-HCl, pH 8. Cell lysates were kept for 10 min on ice followed by centrifugation at 20,000 g for 10 min at 4°C. Cell lysates were then boiled at 95°C for 5 minutes. Total protein (90 μg) was then electrophoresed on a 12.5% SDS-PAGE gel and transferred overnight onto nitrocellulose paper. Western blotting was performed using a goat polyclonal anti-mouse Lipocalin-2/NGAL antibody (R&D Systems, MN, USA) and revealed using an HRP-conjugated donkey antibody to goat IgG (Santa Cruz Biotechnology, Santa Cruz, CA). Bands were visualized using SuperSignal West Pico Chemiluminescent Substrate (Thermo Scientific, IL USA).

## Results

The transcriptional kinetics of *Lcn2 *follows that of the UPR in murine prostate cancer cells

**Figure 1:**
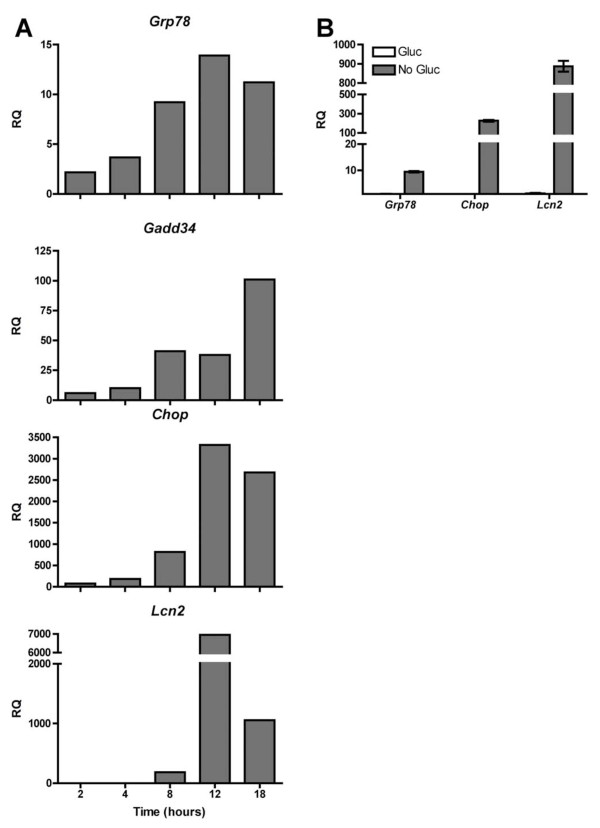
***Lcn2 *and pro-inflammatory cytokine gene transcription in murine prostate cancer cells follows the kinetics of the UPR**. **(A) **TC1 cells were treated with Tg (300 nM) for the indicated times, after which mRNA was isolated and analyzed by RT-qPCR for markers of UPR activation and *Lcn2 *transcription. Data columns indicate the relative quantification (RQ), or fold difference, in transcript level between Tg- and vehicle-treated TC1 cells. A representative experiment of at least three independent experiments per time point is shown. **(B) **TC1 cells were cultured in RPMI lacking glucose for 24 h and analyzed by RT-qPCR for markers of UPR activation and *Lcn2 *transcription. Error bars represent SEM of 2 biological replicates representative of at least 3 independent experiments. Statistical analysis was performed using an unpaired two-tailed *t *test (**p < 0.01; ***p < 0.001).

We first suspected a link between Lcn2 and tumor cell ER stress when we found that among the genes most upregulated in murine A20 lymphoma cells following treatment with thapsigargin (Tg), a canonical inducer of ER stress, was Lcn2 [19] (Additional File 1 Table S1). Because of this observation, we proceeded to investigate the effect of ER stress on murine prostate cancer cells from TRAMP mice, a transgenic model of spontaneous prostate adenocarcinoma that recapitulates the progression, genetic changes, and histology of human prostate cancer [20]. TRAMP C1 (TC1) cells are derived from TRAMP mice and form tumors when injected orthotopically [21]. Tg-treated TC1 cells upregulated *Lcn2 *transcription following kinetics that closely match those of three UPR genes, *Grp78*, *Gadd34*, and *Chop*, peaking between 12 and 18 hours after Tg treatment (Figure 1A). Physiological ER stress induced in TC1 via glucose deprivation also caused *Lcn2 *transcriptional upregulation (Figure 1B). We confirmed that cancer cells of diverse histological origin, including murine melanoma and lung carcinoma, and human colon, ovarian, and breast carcinoma, upregulate *LCN2 *transcription under ER stress (Additional File 2 Fig. S1). Pharmacological ER stress also induces *LCN2 *transcription in non-neoplastic cells, although more robustly in cells of epithelial versus myeloid origin (Additional File 3 Fig. S2). These results suggest that *Lcn2 *transcription may lie downstream of UPR activation.

### Lcn2 upregulation is UPR-mediated and NF-κB-dependent

**Figure 2:**
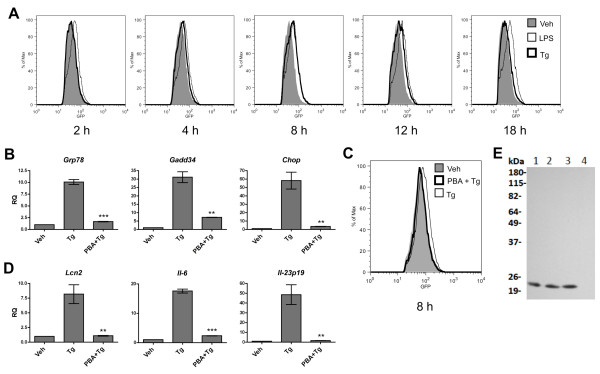
**ER stress-mediated *Lcn2 *transcription is dependent on NF-κB activation in murine prostate cancer cells**. **(A) **TC1.pNGL cells were treated with Tg (300 nM) or vehicle control (Veh), or LPS, for the indicated times. Cells were analyzed for EGFP reporter fluorescence by flow cytometry. Results shown are representative of 2-5 experiments. **(B) **TC1.pNGL cells were treated with Tg with or without 10 mM 4-phenylbutyric acid (PBA), or vehicle only. RNA was isolated and analyzed for UPR activation by RT-qPCR. Data columns indicate the fold difference in transcript level between drug- and vehicle-treated TC1.pNGL cells. Error bars represent SEM of 2-3 biological replicates representative of 3 independent experiments. **(C) **TC1.pNGL cells were treated as in (B) and analyzed for EGFP reporter fluorescence by flow cytometry. Cell viability of all treatment groups was > 99%. **(D)** TC1.pNGL cells from (B) were also analyzed for *Lcn2*, *Il-6*, and *Il-23p19 *transcription by RT-qPCR. Data columns indicate the fold difference in transcript level between drug- and vehicle-treated TC1.pNGL cells, and error bars represent SEM of 2-3 biological replicates representative of 3 independent experiments. Statistical analysis was performed using an unpaired two-tailed *t *test (**p < 0.01; ***p < 0.001). **(E) **Untreated TC1 cells (lane 1) or TC1 treated with vehicle control (lane 2), Tg (lane 3), or Tg + PBA (lane 4), as indicated above, were analyzed for Lipocalin 2 production by Western blot.

We and others have suggested that the response to ER stress can lead to activation of an inflammatory transcriptional program [14,19]. *Lcn2 *transcription has already been shown to be dependent on NF-κB activation in breast and thyroid cancer cells [2,10,11] and NF-κB has been shown to bind to the *Lcn2 *promoter [11]. To test whether transcription of *Lcn2 *is associated with activation of NF-κB in prostate cancer cells, we created a transgenic TC1 cell line, which carries an NF-κB reporter plasmid (TC1.pNGL) containing an enhanced green fluorescent protein (EGFP)-luciferase fusion gene [22]. Induction of ER stress in TC1.pNGL with Tg revealed NF-κB-driven fluorescence between 4 and 12 hours after treatment, with a peak at 8 hours (Figure 2A). Therefore, this result indicates that ER stress triggers NF-κB activation, confirming previous reports [23,24].

Next, we probed TC1 cells using qPCR for transcription of two pro-inflammatory cytokines, *Il-6 *and *Il-23p19*, both of which have been implicated in tumor progression [25,26]. We detected transcriptional upregulation of these two cytokines with kinetics matching that of the UPR genes, *Grp78*, *Gadd34*, and *Chop*, peaking at 18 hours after Tg exposure (Additional File 4 Fig. S3). Thus, we found that NF-κB activation directly precedes the transcriptional activation of *Lcn2 *and that of pro-inflammatory cytokines, suggesting that *Lcn2 *transcription is part of an UPR-mediated inflammatory program in prostate cancer cells.

To verify that the ER stress response is necessary for NF-κB activation and consequent cytokine and *Lcn2 *transcriptional upregulation, we treated TC1.pNGL cells with 4-phenyl butyric acid (PBA), a chemical chaperone known to reduce ER stress [27], during Tg stimulation. PBA-treated cells displayed significant inhibition of the UPR as indicated by decreased levels of *Grp78*, *Gadd34 *and *Chop*, relative to cells treated with Tg alone (Figure 2B). Notably, PBA-treated cells did not activate NF-κB in response to Tg treatment (Figure 2C) and this resulted in a complete abrogation of ER stress-mediated *Lcn2 *transcription, as well as *Il-6 *and *Il-23p19 *transcription (Figure 2D). Western blotting of TC1 treated with Tg alone or in combination with PBA verified the UPR-specific translation of Lcn2 (Figure 2E).

**Figure 3:**
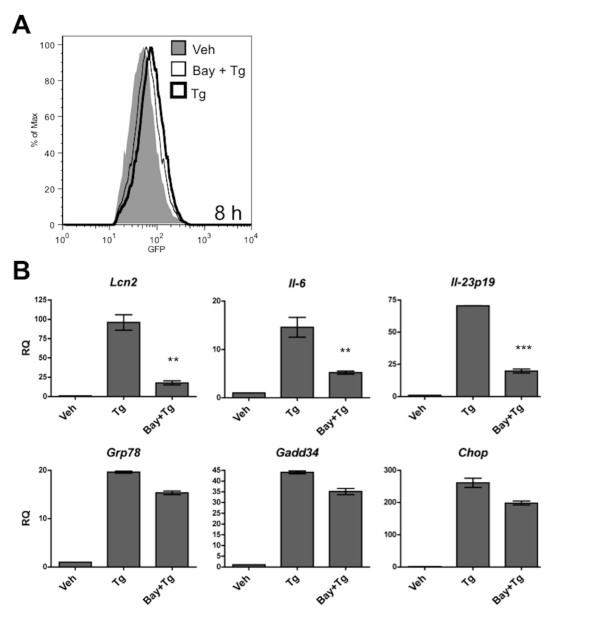
**UPR-mediated *Lcn2 *transcription is NF-κB dependent**. **(A) **TC1.pNGL cells were treated for 8 h with Tg (300 nM) with or without Bay 11-7082 (25 μM), or vehicle alone, and analyzed for EGFP reporter fluorescence by flow cytometry. Cells viability in all treatment groups was > 99%. Results shown are representative of 3 experiments. (MFI Veh: 50; MFI Bay + Tg: 61.8; MFI Tg: 72.7) **(B) **RNA was isolated from cells from (A) and analyzed for UPR activation and *Lcn2*, *Il-6*, and *Il-23p19 *transcription by RT-qPCR. Data columns indicate the fold difference in transcript level between drug- and vehicle-treated TC1.pNGL cells. Error bars represent SEM of 2-3 biological replicates. Statistical analysis was performed using an unpaired two-tailed *t *test (**p < 0.01; ***p < 0.001).

To confirm that the UPR drives the transcriptional upregulation of *Lcn2 *via NF-κB, we treated Tg-activated TC1.pNGL cells with BAY 11-7082, an irreversible inhibitor of IκBα phosphorylation, which results in the down-regulation of NF-κB activation [28]. At the dose used (25 μM), BAY 11-7082 inhibited ER stress-mediated NF-κB activation by 48% (MFI Veh: 50; MFI Bay + Tg: 61.8; MFI Tg: 72.7) (Figure 3A) with minimal effect on the UPR and cell viability (all treatment groups had > 99% viability) (Figure 3B). At higher concentrations of Bay 11-7082, UPR-induced NF-κB activation was more greatly inhibited but cell death and confounding off-target effects (e.g., UPR inhibition) were also increased dramatically. Consistent with our prediction, BAY 11-7082 markedly inhibited the UPR-induced transcriptional upregulation of *Lcn2*, *Il-6 *and *Il-23p19 *(Figure 3B). Taken together, these data indicate that ER stress in murine prostate cancer cells drives the NF-κB-dependent transcription of *Lcn2 *and pro-inflammatory cytokines.

### Human prostate cancer cells upregulate *LCN2 *and pro-inflammatory cytokines in response to ER stress

**Figure 4:**
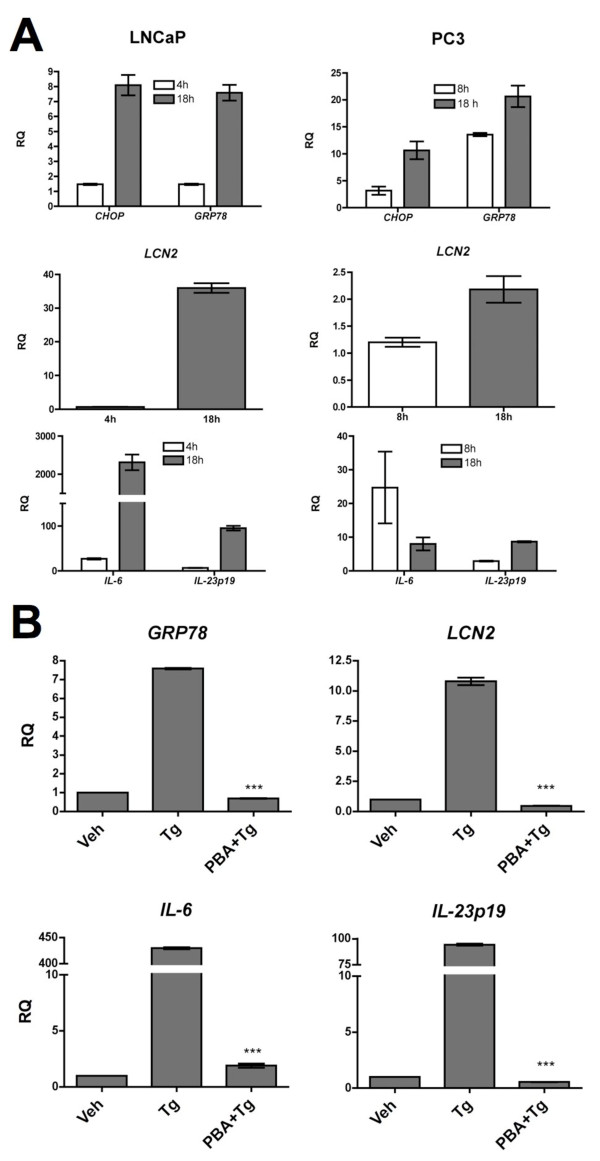
**The ER stress response drives *LCN2 *transcription in human prostate cancer cells**. **(A) **LNCaP or PC3 cells were treated with Tg (300 nM) for the indicated times. RNA was isolated and analyzed for UPR activation, *LCN2*, *Il-6*, and *Il-23p19 *transcription by RT-qPCR. Data columns indicate the fold difference in transcript level between drug- and vehicle-treated, or untreated cells and error bars represent SEM of 2 biological replicates representative of three independent experiments. **(B) **LNCaP cells were treated for 18 h with Tg alone, PBA (10 mM) and Tg, or vehicle only. RNA was isolated and analyzed for UPR activation, *LCN2*, *IL-6*, and *IL-23p19 *transcription by RT-qPCR. Data columns indicate the fold difference in transcript level between drug- and vehicle-treated cells and error bars represent SEM of 2-3 biological replicates representative of two independent experiments. Statistical analysis was performed using an unpaired two-tailed *t *test (***p < 0.001).

To extend these findings to human prostate cancer, we interrogated *LCN2 *activation during conditions of ER stress in two human prostate cancer cell lines, LNCaP and PC3. We found that *LCN2 *is upregulated by Tg-induced ER stress in both cell lines, as were the pro-inflammatory cytokines, *IL-6 *and *IL-23p19 *(Figure 4A). LNCaP cells also upregulated *LCN2*, *IL-6 *and *IL-23p19 *transcription in response to treatment with tunicamycin, another inducer of ER stress, albeit to a lower magnitude than cells treated with Tg (Additional File 5 Fig. S4). Corroborating our results in TC1 cells, treatment of LNCaP cells with PBA prevented Tg-induced *LCN2*, *IL-6 *and *IL-23p19 *transcription (Figure 4B). Taken together, these results indicate that the ER stress response drives transcription of *LCN2 *and pro-inflammatory cytokines in human prostate cancer cells.

## Discussion

Because *Lcn2 *is a gene found in non-neoplastic cells that has been shown to function in the regulation of tumor cell growth and progression [3] it fulfills the canonical definition of a proto-oncogene [29]. Here we establish a link between the ER stress response in prostate cancer cells and Lcn2 upregulation. Evidence is also provided that this event may be part of a larger program of an NF-κB-dependent response. Lastly, we show that diminishing the magnitude of the UPR using a chemical chaperone can dramatically decrease the production of Lcn2 and tumorigenic cytokines. The present report finds indirect corroboration in earlier studies showing that stearoyl-CoA desaturase 1 (SCD1)-deficient mice fed a very low-fat diet experience ER stress in the liver concomitant with high levels of *Lcn2 *transcription [30], and that IRE1α or PERK can activate NF-κB [24,31], which itself binds the *Lcn2 *promoter, driving its transcription [11]. Our findings are also corroborated by the observation that the *Lcn2 *promoter contains binding sites the ER stress-responsive transcription factor C/EBP (CCAAT/enhancer-binding protein) [11,32].

The link between ER stress and cancer progression has been previously suggested. For instance, *Grp78 *hemizygous (+/-) mice crossed with MMTVPyVT heterozygous transgenic mice display significantly decreased tumor proliferation, survival, and angiogenesis [33]. Similarly, the inactivation of ER stress signaling by mutations of PERK, or by a dominant-negative PERK, in tumor cells, results in tumors that are smaller and less aggressive than their normal counterparts when implanted into mice [34]. Furthermore, increasing levels of Grp78 positively correlate with solid tumor progression and poor clinical outcome [17,35]. In parallel, a link between Lipocalin 2 and cancer has also been reported. For instance, Lipocalin 2 is upregulated in human cancers of several origins and, importantly, its levels correlate with the aggressiveness of clonally-derived murine breast cancer cell lines [2].

In light of the present findings, it is tempting to speculate that Lipocalin 2 expression levels correlate with tumor progression because of the concomitant upregulation of the UPR. In the case of prostate cancer, it will be interesting to simultaneously analyze other markers of prostate cancer progression, *e.g*., TMPRSS-ERG fusion events [36], to see if their presence also correlates with upregulation of Lipocalin 2. If our prediction turns out to be correct, we anticipate that Lipocalin 2 could serve as a novel biomarker of prostate cancer progression [18].

What signaling elements connect the UPR to NF-κB activation and Lipocalin 2 upregulation in cancer cells? Fu et al. [16] demonstrated that prostate-specific knockdown of *Grp78 *in *Pten*-null mice abrogates AKT phosphorylation (and tumor development), and that human prostate cancer cells under ER stress increase AKT phosphorylation in a Grp78-dependent manner. Recently, it was shown that inhibition of AKT phosphorylation and/or NF-κB activation both caused downregulation of LCN2 expression in human breast cancer cells [2]. Taken together, these findings suggest that ER stress in cancer cells may be the primary triggering event for Lipocalin 2 activation via the Grp78/AKT/NF-κB axis. The recently demonstrated efficacy of anti-Lcn2 antibody therapy in slowing mammary tumor growth and metastasis in mice [2] together with the potential use of small molecules and chemical chaperones to inhibit Grp78 and UPR [27,37], identifies the ER stress-Lipocalin 2 axis as a novel target for synergistic therapeutic intervention in several cancers, including prostate cancer.

## Conclusions

Here, we demonstrate that the UPR evoked by pharmacological and physiological ER stress in prostate cancer cells can drive the transcription and translation of the proto-oncogene, *Lcn2*. This process is mediated by UPR-dependent activation of NF-κB.

## Abbreviations

Chop: CCAAT/enhancer-binding protein homologous protein; EGFP: Enhanced Green Fluorescent Protein; ER: Endoplasmic Reticulum; Gadd34: Growth Arrest and DNA-Damage Inducible Protein; Grp78: 78 kDa Glucose-Regulated Protein; IkBα: Nuclear Factor of kappa Light Polypeptide Gene Enhancer in B-cells Inhibitor, alpha; Il-6: Interleukin 6; Il23-p19: Interleukin 23, p19 subunit; Lcn2: Lipocalin 2; LNCaP: Lymph Node Carcinoma of the Prostate; NF-κB: Nuclear Factor of kappa Light Polypeptide Gene Enhancer in B cells; PBA: 4-phenylbutyric acid; PC3: Prostate Cancer Cell Line 3; Tg: Thapsigargin; TRAMP: Transgenic Adenocarcinoma of the Mouse Prostate; UPR: Unfolded Protein Response.

## Competing interests

The authors declare that they have no competing interests.

## Authors' contributions

NRM designed and performed the experiments, analyzed the data, and wrote the manuscript. JR performed experiments and analyzed the data. GA created the TC1.pNGL cell line and performed experiments. AFP performed experiments. MCW performed the initial experiments. MZ helped design experiments and wrote the manuscript. All authors read, edited, and approved the final manuscript.

## Pre-publication history

The pre-publication history for this paper can be accessed here:

http://www.biomedcentral.com/1471-2407/11/229/prepub

## Supplementary Material

Additional file 1 **Table S1**. Selected gene expression profiling in A20 B lymphoma cells during ER stress. Click here for file

Additional file 2 **Figure S1**. ER stress in mouse and human neoplastic cells evokes *Lcn2 *transcription. Click here for file

Additional file 3 **Figure S2**. ER stress in non-neoplastic cells of mouse and human origin induces *Lcn2 *transcription. Click here for file

Additional file 4 **Figure S3**. ER stress in prostate cancer cells promotes transcription of proinflammatory cytokines. Click here for file

Additional file 5 **Figure S4**. Tunicamycin-induced ER stress activates *Lcn2 *transcription in human prostate cancer cells. Click here for file
