# Supplementary material for: ER stress drives Lipocalin 2 upregulation in prostate cancer cells in an NF-κB-dependent manner
Source: BMC Cancer. 2011 Jun 7;11:229. doi: 10.1186/1471-2407-11-229 (PMC3146445; doi:10.1186/1471-2407-11-229)
Supplement: Additional file 1 — Table S1. Selected gene expression profiling in A20 B lymphoma cells during ER stress. [file 1471-2407-11-229-S1.DOC]

**Supplementary Table 1**

Selected gene expression profiling in A20 B lymphoma cells during ER stress

| **Fold Increase in Expression**  **(8 h / 24 h)** | **Gene Product (Symbol)** | **Gen Bank**  **Accession No.** | **Function** |
| --- | --- | --- | --- |
| **12.978 / 13.188** | DNA-damage inducible transcript 3 (*Ddit3, Chop)* | NM_007837.2 | UPR |
| **4.720 / 6.883** | myeloid differentiation primary response gene 116 (*Myd116*, *Gadd34*) | NM_008654.1 | UPR |
| **2.328 / 4.455** | heat shock 70 kD protein 5 (glucose-regulated protein) (*Hspa5, BiP***)** | NM_022310.2 | UPR |
| **2.606 / 43.674** | lipocalin 2 (*Lcn2*) | NM_008491.1 | Tumorigenesis |
| **7.842 / 17.787** | matrix metalloproteinase 13 (*Mmp13*) | NM_008607.1 | Inflammation |
| **-0.102 / 16.461** | nuclear protein 1 (*Nupr1*) | NM_019738.1 | Tumorigenesis |
| **18.073 / 6.584** | interleukin 23, alpha subunit p19 (*Il23a*) | NM_031252.1 | Inflammation |
| **2.697 / 2.197** | CCAAT/enhancer binding protein (C/EBP), beta (*Cebpb*) | NM_009883.1 | Inflammation |
| **2.170 / 0.236** | interleukin 6 (*Il6*) | NM_031168.1 | Inflammation |
