# Supplementary material for: ER stress drives Lipocalin 2 upregulation in prostate cancer cells in an NF-κB-dependent manner
Source: BMC Cancer. 2011 Jun 7;11:229. doi: 10.1186/1471-2407-11-229 (PMC3146445; doi:10.1186/1471-2407-11-229)
Supplement: Additional file 2 — Figure S1. ER stress in mouse and human neoplastic cells evokes Lcn2 transcription. [file 1471-2407-11-229-S2.PDF]

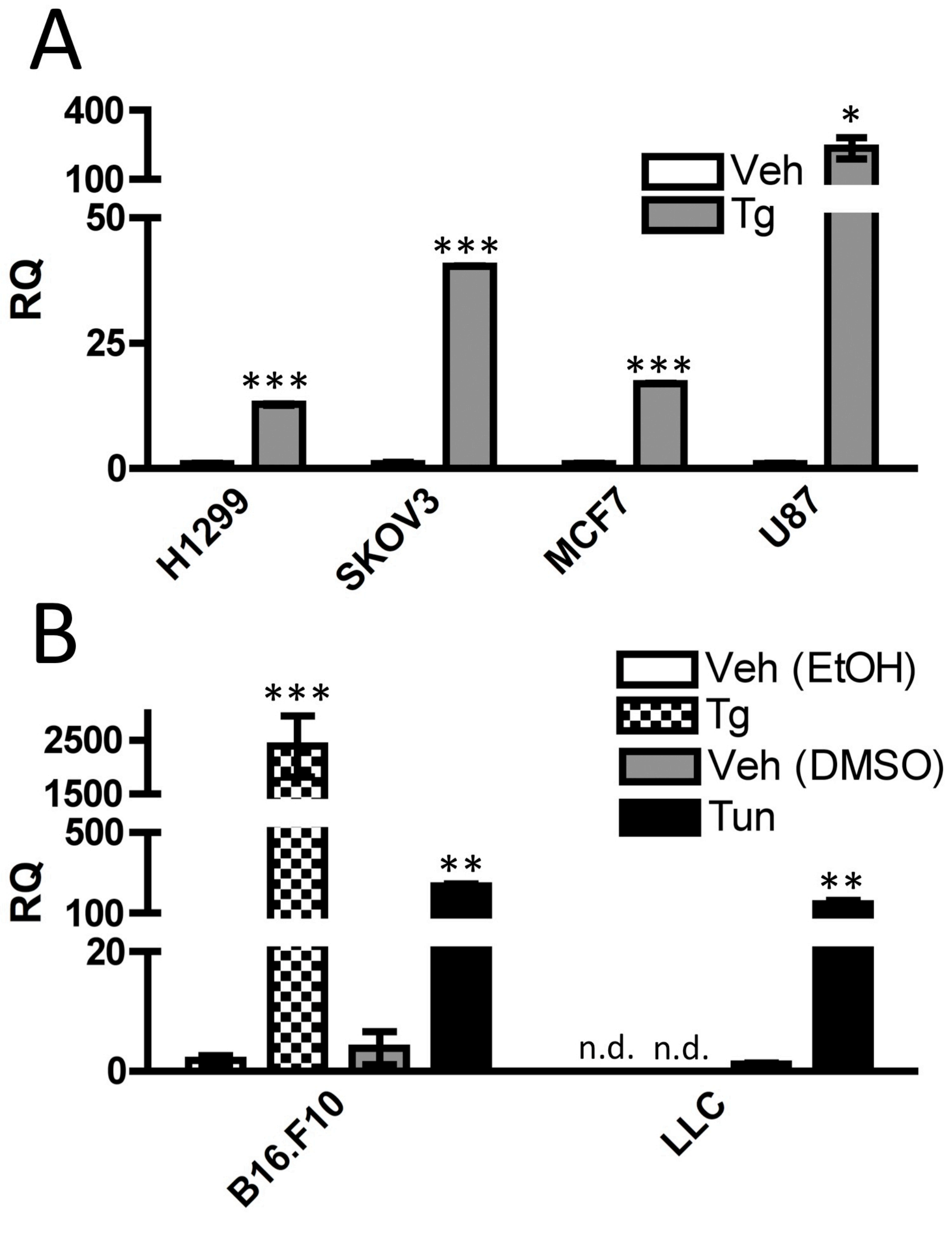

Figure S1. ER stress in mouse and human neoplastic cells evokes *Lcn2* transcription.

(A) Human cancer cells were treated with Tg (300 nM) for 18 h and assayed for *LCN2* transcription by RT-qPCR. Data columns indicate the fold difference in transcript level between Tg- and vehicle-treated cells. Error bars represent the SD from a single experiment. Data is representative of two independent experiments. Statistical analysis was performed using an unpaired two-tailed *t* test (*p < 0.05; **p < 0.01; ***p < 0.001). **(B)** Murine cancer cells were treated with Tg (300 nM) or Tun (5 μg/mL) for 18 h and assayed for *Lcn2* transcription by RT-qPCR. Columns indicate the fold increase in transcript level (RQ) of each treatment group. The value of a vehicle control (EtOH/DMSO) was set arbitrarily to 1. Error bars represent SEM of 2 biological replicates representative of at least 2 independent experiments. n.d. = not determined.
