# Supplementary material for: ER stress drives Lipocalin 2 upregulation in prostate cancer cells in an NF-κB-dependent manner
Source: BMC Cancer. 2011 Jun 7;11:229. doi: 10.1186/1471-2407-11-229 (PMC3146445; doi:10.1186/1471-2407-11-229)
Supplement: Additional file 3 — Figure S2. ER stress in non-neoplastic cells of mouse and human origin induces Lcn2 transcription. [file 1471-2407-11-229-S3.PDF]

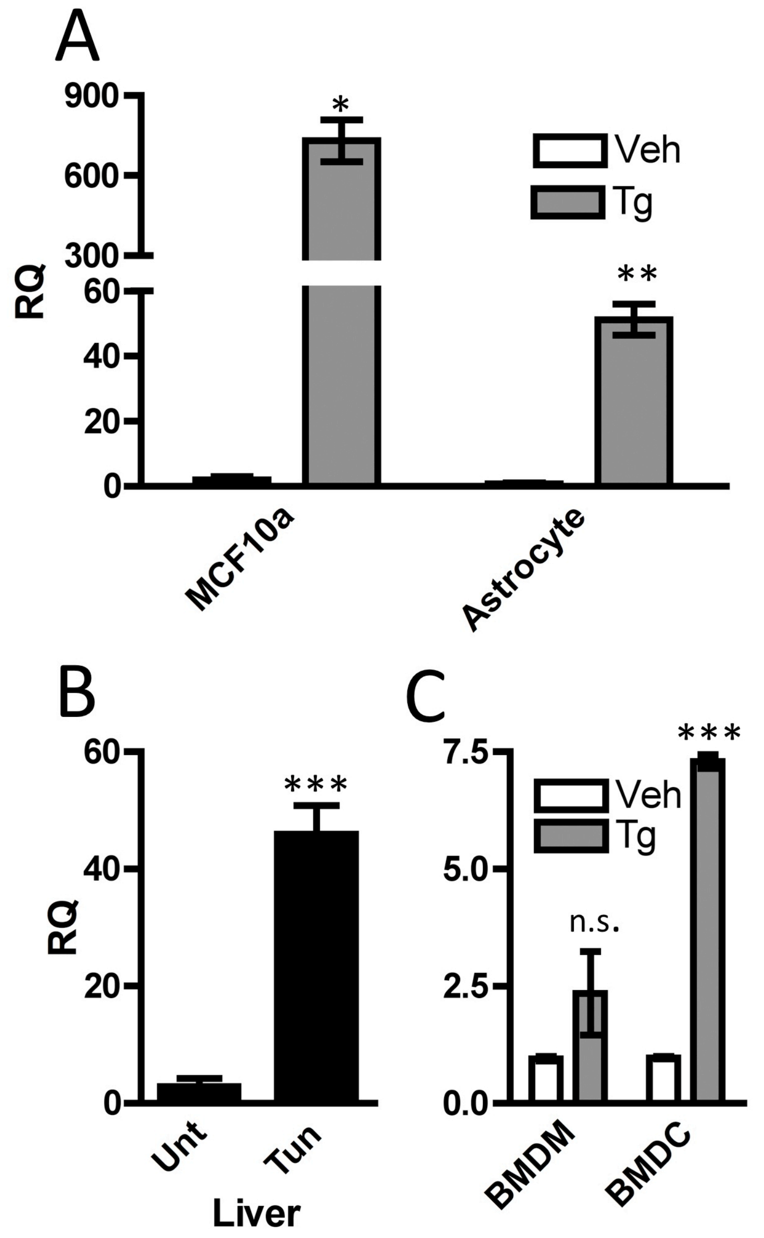

Figure S2. ER stress in non-neoplastic cells of mouse and human origin induces *Lcn2* transcription.

(A) Human cells of non-neoplastic origin were treated with Tg (300 nM) for 18 h and assayed for *Lcn2* transcription by RT-qPCR. Error bars represent SEM of 2 biological replicates. **(B)** C57/BL6 mice (n=4) were injected i.p. with 2 mg/kg of tunicamycin or an equal volume of vehicle (PBS). After 8 h, livers were harvested and assayed for *Lcn2* transcription by RT-qPCR. Error bars represent SEM. **(C)** Murine bone marrow-derived macrophages (BMDM) or dendritic cells (BMDC) were treated with Tg (300 nM) for 24 h and assayed for *Lcn2* transcription by RT-qPCR. Error bars represent SEM of 2 biological replicates. For all data, columns indicate the fold increase in transcript level (RQ) of each treatment group. The value of a vehicle control was set arbitrarily to 1. Statistical analysis was performed using an unpaired two-tailed *t* test (*p < 0.05; **p < 0.01; ***p < 0.001, n.s. = non-significant).
