# Supplementary material for: ER stress drives Lipocalin 2 upregulation in prostate cancer cells in an NF-κB-dependent manner
Source: BMC Cancer. 2011 Jun 7;11:229. doi: 10.1186/1471-2407-11-229 (PMC3146445; doi:10.1186/1471-2407-11-229)
Supplement: Additional file 4 — Figure S3. ER stress in prostate cancer cells promotes transcription of proinflammatory cytokines. [file 1471-2407-11-229-S4.PDF]

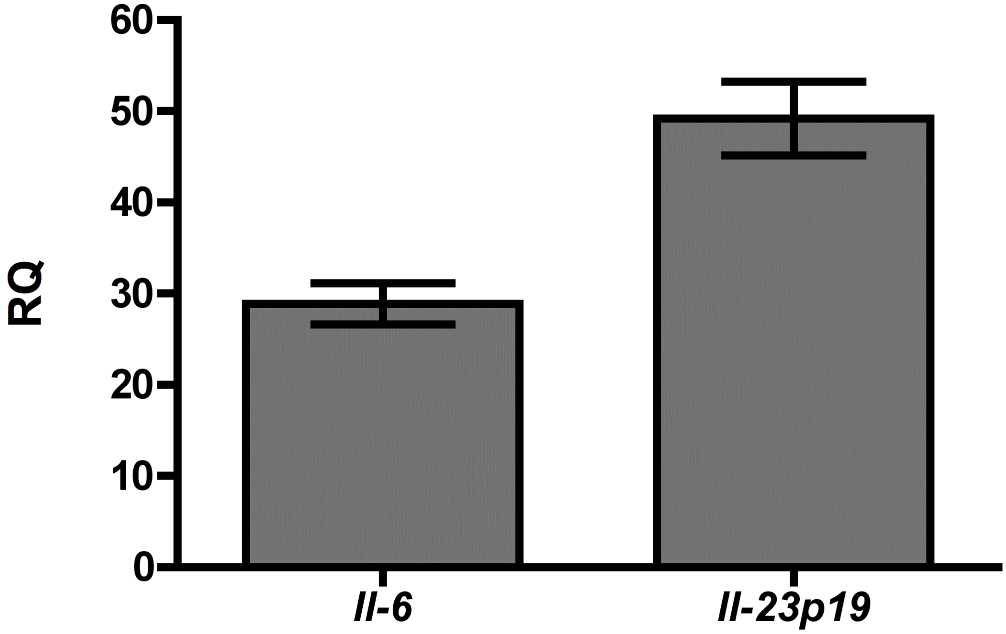

Figure S3. ER stress in prostate cancer cells promotes transcription of proinflammatory cytokines.

TC1 cells were treated with Tg (300 nM) for 18 h and assayed for *Il-6* and *Il-23p19* transcription by RT-qPCR. Data columns indicate the fold difference in transcript level between Tg- and vehicle-treated TC1 cells. Error bars represent SEM of 2 biological replicates representative of at least 3 independent experiments.
