# Supplementary material for: ER stress drives Lipocalin 2 upregulation in prostate cancer cells in an NF-κB-dependent manner
Source: BMC Cancer. 2011 Jun 7;11:229. doi: 10.1186/1471-2407-11-229 (PMC3146445; doi:10.1186/1471-2407-11-229)
Supplement: Additional file 5 — Figure S4. Tunicamycin-induced ER stress activates Lcn2 transcription in human prostate cancer cells. [file 1471-2407-11-229-S5.PDF]

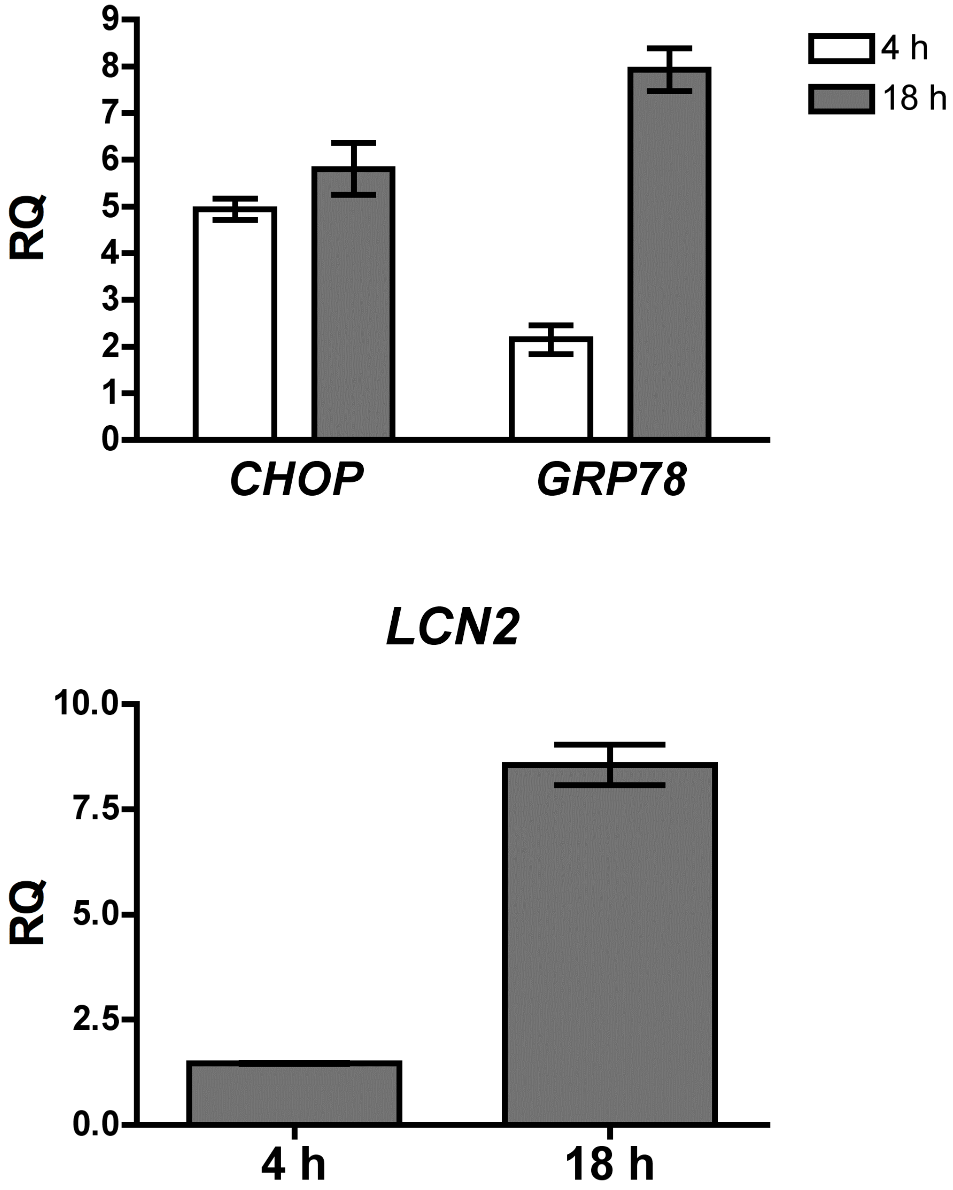

Figure S4. Tunicamycin-induced ER stress activates *Lcn2* transcription in human prostate cancer cells.

LNCaP cells were treated with tunicamycin (5 μg/mL) for the indicated times, after which mRNA was isolated and analyzed by RT-qPCR for markers of UPR activation and *Lcn2* transcription. Data columns indicate the fold difference in transcript level between tunicamycin- and vehicle (DMSO)-treated LNCaP cells, whose gene expression was set arbitrarily to 1. Error bars represent SEM of 2 biological replicates.
